# The Impact of KLF2 Modulation on the Transcriptional Program and Function of CD8 T Cells

**DOI:** 10.1371/journal.pone.0077537

**Published:** 2013-10-14

**Authors:** Gavin C. Preston, Carmen Feijoo-Carnero, Nick Schurch, Victoria H. Cowling, Doreen A. Cantrell

**Affiliations:** 1 Department of Cell Signalling & Immunology, College of Life Sciences, University of Dundee, Dundee, United Kingdom; 2 Data Analysis Group, Department of Biological Chemistry and Drug Discovery, College of Life Sciences, University of Dundee, United Kingdom; Oklahoma Medical Research Foundation, United States of America

## Abstract

Krüppel-like factor 2 (KLF2) is a transcription factor that is highly expressed in quiescent T lymphocytes and downregulated in effector T cells. We now show that antigen receptor engagement downregulates KLF2 expression in a graded response determined by the affinity of T cell antigen receptor (TCR) ligand and the integrated activation of protein kinase B and the MAP kinases ERK1/2. The present study explores the importance of KLF2 downregulation and reveals that the loss of KLF2 controls a select portion of the CD8 effector T cell transcriptional program. In particular, KLF2 loss is required for CD8 T cells to express the inflammatory chemokine receptor CXCR3 and for maximum clonal expansion of T cells. KLF2 thus negatively controls the ability of CD8 T cells to respond to the CXCR3 ligand CXCL10. Strikingly, the KLF2 threshold for restraining expression of CXCR3 is very low and quite distinct to the KLF2 threshold for restraining T cell proliferation. KLF2 is thus an analogue (tunable) not a digital (on/off) cellular switch where the magnitude of KLF2 expression differentially modifies the T cell responses.

## Introduction

Krüppel-like factor 2 (KLF2) is a transcription factor that can control stem cell self renewal, the inflammatory properties of the endothelium and lymphocyte trafficking[1–5]. In T lymphocytes, KLF2 is highly expressed in naïve and memory T cells but only expressed at low levels in effector T cells such as cytotoxic T lymphocytes (CTL)[6–8]. The loss of KLF2 by effector T cells reflects that KLF2 expression is rapidly downregulated in response to triggering of the T cell antigen receptor (TCR)[9]. This downregulation is then reinforced or modulated by members of the common cytokine receptor gamma-chain (γc) family of cytokines. For example, Interleukin 2 (IL-2), which promotes CTL differentiation, can sustain KLF2 downregulation[7,10,11]. It was originally proposed that KLF2 functioned to regulate T cell quiescence by downregulating expression of *c-myc*[12] whilst inducing expression of the cell cycle inhibitor *p21* ^*cip1*^[13]. This view has been challenged recently by the realisation that a primary role for KLF2 in naïve T cells is to control expression of CD62L, an adhesion receptor essential for T cell transmigration from the blood into secondary lymphoid tissues, and S1P1, a lysosphingolipid receptor that controls T cell egress from thymic and peripheral lymphoid tissue[14–16]. KLF2 thus controls T cell trafficking between the blood and lymphoid organs[3,16,17]. KLF2 has also been proposed to suppress expression of inflammatory chemokine receptors such as CXCR3[4]. However, the control of CXCR3 by KLF2 in thymocytes is not cell autonomous but rather a consequence of unrestrained cytokine production. Accordingly, the T cells that develop in the absence of KLF2 overproduce Interleukin 4 (IL-4), Interferon gamma (IFNγ) and Tumour Necrosis Factor alpha (TNFα), which promotes upregulation of inflammatory chemokine receptors on thymocytes[17]. The loss of KLF2 is thus a key part of the transcriptional program that directs the trafficking of activated T cells away from lymphoid tissue and promotes their extravasation to peripheral tissues. Moreover, KLF2 is required to repress cytokine production by naïve T cells[17]. 

The importance of KLF2 for T cells highlights the importance of understanding both the intracellular signals that regulate KLF2 expression and the relevance and full consequences of KLF2 downregulation in TCR triggered CD8 T cells. There are a number of unanswered questions. Is KLF2 loss essential for inflammatory cytokine expression in activated CD8 T cells? Is KLF2 downregulation required for CD8 T cells to proliferate? Another issue that has not been explored is how the T cell antigen receptor controls KLF2 expression. One relevant fact is that KLF2 expression in T cells is controlled by Forkhead box transcription factors (FoxOs)[18,19]. Moreover, in effector CTL the cytokine IL-2 sustains KLF2 downregulation by activating the serine/threonine kinase PKB which then phosphorylates FoxOs causing their nuclear exclusion and hence terminating the expression of FoxO regulated genes such as KLF2[18,20–22]. PKB is activated in response to stimuli that activate phosphoinositide 3-kinases (PI3Ks) and increase cellular levels of phosphatidylinositol 3,4,5-trisphosphate (PI[3–5]P_3_). In this context, KLF2 levels are downregulated in T cells that constitutively activate PI[3–5]P_3_/PKB mediated signalling due to loss of the tumour suppressor Phosphatase and Tensin Homolog (PTEN)[21,23,24]. It has not, however, been determined whether the ability of TCR engagement to rapidly terminate KLF2 expression is controlled by PKB or whether other TCR mediated signals are involved.

Accordingly, the object of the present study was to explore further the regulation and function of KLF2 in CD8 T cells as they respond to immune stimuli. The experiments reveal KLF2 expression in T cells is dynamic and determined by both the strength and duration of antigen receptor or cytokine signalling. Key insights from these studies include that TCR mediated activation of both PKB and the MAP kinases ERK1/2 is required for KLF2 loss. Transcriptional profiling revealed that loss of KLF2 is essential for T cells to acquire the full effector T cell transcriptional program. One key observation is that KLF2 loss is essential for the TCR to upregulate expression of the inflammatory chemokine receptor CXCR3. The ability of KLF2 to negatively regulate expression of CXCR3 reveals that the role of KLF2 in controlling CD8 T cell trafficking is not limited to its role as a positive regulator of CD62L and S1P1. The data also reveal different KLF2 thresholds for the regulation of T cell proliferation versus trafficking. KLF2 thus controls an analogue and not a digital response allowing differences in the magnitude of KLF2 expression to fine tune CD8 T cell fate.

## Materials and Methods

### Ethics Statement

All mice used were bred and maintained under specific pathogen-free conditions in the Biological Resource Unit at the University of Dundee. The procedures used were approved by the University Ethical Review Committee and authorised by a project licence under the UK Home Office Animals (Scientific Procedures) Act 1986.

### Mice and cell culture

Mice used were P14 TCR transgenic mice with a TCR recognising the gp33-41 peptide of LCMV (KAVYNFATM) and OT-1 transgenic mice, which express a TCR recognising the ovalbumin derived peptide SIINFEKL. Naïve CD8 T cell were negatively selected from lymph nodes using AutoMacs (Miltenyi Biotechnology). 1ng/milliliter of SIINFEKL or 10ng/ml of the altered peptide ligands (SIIQFEKL, SIIQFEHL, SIIQFERL), were used for OT-1 TCR triggering. P14 CTL were produced by activating splenocytes for 2 days with 100ng/ml gp33-41 peptide, then washed and cultured with IL-2 (20ng/ml, Novartis) for a further 3-5 days unless otherwise stated. Where indicated, rapamycin (Calbiochem) was used at 20nM.

### Retroviral transduction

The GFP control plasmid construct was made by PCR amplification of EGFP from a pEGFP construct (Clontech) and cloning into the pBMN-LZRS vector (Addgene) as a *Hind*III/*Not*I fragment, replacing the l*acZ* gene. KLF2 cDNA was PCR amplified and cloned into pEGFP-C1 as a *Hind*III/*BamH*I fragment. The GFP-KLF2 construct was made by PCR amplification of EGFP-KLF2 and cloning into pBMN-LZRS as an *EcoR*I fragment, replacing the *lacZ* gene. The GFP-FoxOAAA has been previously described[25]. Phoenix ecotropic packaging cells[26] were transfected with plasmid using calcium phosphate transfection. Virus was harvested and used to transduce T cells as previously described[22]. Transduced activated CD8 T cells were generated by activation with gp33-41 peptide, retroviral transduction at 18 hours post activation, washing at 48 hours post activation and followed by 2 days culture with IL-2 in all experiments.

### Flow Cytometry and Cell Sorting

Cell counts were performed with Caltag counting beads (Invitrogen) according to the manufacturer’s instructions. The following antibodies were used for staining: CD8-FITC, CD62L-APC (BD Pharmingen) and CXCR3-PerCPCy5.5 (eBiosciences). Cellular DNA content was measured using Hoechst 33342 (Molecular Probes). DNA synthesis was measured using the Clickit-EdU kit (Invitrogen) according to the manufacturer’s instructions. CTL were set up at 5x10^5^/ml and incubated with 10µM EdU for 30 minutes before fixation and staining. Data were collected on FACS Calibur and LSR Fortessa machines (Beckton Dickinson) and analyzed using FlowJo software (Treestar). Fluorescence Activated Cell Sorting (FACS) was performed on a FACS Vantage Cell Sorter (Beckton Dickinson).

### Real-Time PCR

RNA was extracted from CTL or naïve cells using the RNeasy Minikit (Qiagen) and used to make cDNA using the cDNA synthesis kit (Quanta Biosciences). Quantitative real time PCR was performed on an iQ5 (Bio-Rad) using SYBR Green Fastmix (Quanta Biosciences). Results were normalised to expression of HPRT or CD8. For KLF2 quantification in naive and retrovirally transduced cells a standard cDNA of known concentration was used to generate a standard curve against which samples were measured. Primers used were as follows: KLF2 forward 5’-TGTGAGAAATGCCTTTGAGTTTACTG-3’, reverse 5’-CCCTTATAGAAATACAATCGGTCATAGTC-3’, KLF2 cDNA forward 5’-GCGCTCTGACGAGCTTACC-3’, reverse 5’-ACATGTGTCGCTTCATGTGC-3’, CD62L forward 5’-ACGGGCCCCAGTGTCAGTATGTG-3’, reverse 5’-TGAGAAATGCCAGCCCCGAGAA-3’, S1P1 forward 5’-GTGTAGACCCAGAGTCCTGCG-3’, reverse 5’-AGCTTTTCCTTGGCTGGAGAG-3’, CXCR3 forward 5’-GCCAAGCCATGTACCTTGAG-3’, reverse 5’-GTCAGAGAAGTCGCTCTCG-3’, Il6ra forward 5’-GTCACGGGCACTCCTTGGATAG-3’, reverse 5’-AGGAATGTGGGCAGGGACATGG-3’, Serpinb9 forward 5’-CAGATGAGGGTGTGGACCTCAG-3’, reverse 5’-TCCCAAGCGCTGAAACAGAGAC-3’, T-bet forward 5’-GATCATCACTAAGCAAGGACG-3’, reverse 5’-GGTCCACCAAGACCACATC-3’, Perforin forward 5’-CGTCTTGGTGGGACTTCAG-3’, reverse 5’-GCATTCTGACCGAGTGGCAG-3’, Interferon gamma forward 5’-TTACTGCCACGGCACAGTC-3’, reverse 5’-AGATAATCTGGCTCTGCAGG-3’, c-myc forward 5’-CCACCAGCAGCGACTCTG-3’, reverse 5’-GAGATGAGCCCGACTCCG-3’, HPRT forward 5’-TGATCAGTCAACGGGGGACA-3’, reverse 5’-TTCGAGAGGTCCTTTTCACCA-3’, CD8 forward 5’-GATATAAATCTCCTGTCTGCCCATC-3’, reverse 5’-ATTCATACCACTTGCTTCCTTGC-3’

### Chromatin Immunoprecipitation

Chromatin Immunoprecipitation was performed as previously described[25] using anti-Pol II (N-20, Santa-Cruz Biotechnology) antibody or normal rabbit IgG (Cell Signalling). The immunocomplexes were collected using chip Grade Protein G Magnetic beads (Cell Signalling) in presence of 0.2mg/ml BSA. Immunoprecipitated chromatin was amplified by Real-Time PCR as above. The primers used were as follows: 

KLF2 upstream promoter (-1500bp upstream TSS) forward 5’-TCAGCCTCACATTCTGCCTC-3’, reverse 5’- GCAGAAACATTGGCGAACTAC-3’

#### KLF2 TSS, forward 5’-**CCACAGCACACACAGTCC**-3’; reverse 5’-**CACGGGCTGGCGAAAGTG**-3’

### Microarray samples and analysis

RNA was prepared as above from cells purified by FACS and samples were forwarded to the Finnish Microarray and Sequencing Center (Turku) for hybridization on Affymetrix 3’ IVT expression chips (mouse genome 430 2.0). The data were normalised and expression measures computed using the Robust Multiarray Average (RMA) method[27]. Hierarchical clustering of the normalised intensity probe-sets confirmed that the biological replicates accurately reflected distinctly identifiable data groups. Simple inter-replicate expression plots, where probe intensities for a given replicate are plotted against the probe intensities for the other replicates in the group, were used to ensure that no large scale systematic effects are present in the data. A linear model was fitted to the normalised intensity data with fixed constants for each replicate group using the R package ‘limma’.[28] P-values for the fold-changes calculated from the linear model are calculated by shrinkage of the empirical Bayes moderated t-statistic for each probe and are then adjusted for multiple hypothesis testing by controlling the False Discovery Rate (using the correction detailed by Benjamini and Hochberg[29]) to produce q-values for linear model analysis. Probe-sets with q-values <0.046% (2σ) were considered to be significantly regulated. Regulation of at least 2-fold was used as a threshold cutoff.

Data are available at: http://www.ncbi.nlm.nih.gov/geo/query/acc.cgi?acc=GSE26568

### Transwell chemotaxis assay

CTL transduced with GFP-KLF2 retrovirus were competitively assayed with co-cultured untransduced CTL for their ability to migrate towards a CXCL10 gradient. Briefly, transwell plates (Corning) were coated with Fibronectin (Sigma) and CXCL10 (R&D Systems) was diluted in media to the relevant concentration. CTL were then added and input controls taken. After 4 hours incubation cell count and GFP percentage were analyzed by flow cytometry and compared to input controls.

### Western Blotting

Cells were lysed at 3x10^7^/ml in 10mM Tris pH7.05, 50mM NaCl, 50mM NaF, 5μM ZnCl_2_, 1mM DTT, 10% glycerol and 0.5% Triton X100 with complete protease inhibitors (Roche). The resultant lysates were separated by SDS-PAGE, transferred onto nitrocellulose membrane and detected by Western blotting using standard techniques. Antibodies used recognised Spi6 (Hycult), GSK3α/β, pT308 PKB, pan PKB, pT24/32FoxO1/3A (Cell Signaling Technology) and pan FoxO1 (Dundee Signal Transduction & Therapeutics).

### Statistical Analyses

Data sets were analysed using SigmaPlot v11.0 (Systat Software Inc., USA). Comparisons between two groups were made using Student’s t test or a non-parametric Wilcoxon Rank-Sum test where appropriate. Comparisons between multiple groups were made using one way analysis of variance (ANOVA) test. Levels of significance are denoted as follows: * p<0.05, ** p<0.01, *** p<0.001. Non-significant results are either not marked or indicated ns.

## Results

### KLF2 expression is determined by the strength of T cell antigen receptor and cytokine signalling

**Figure 1:**
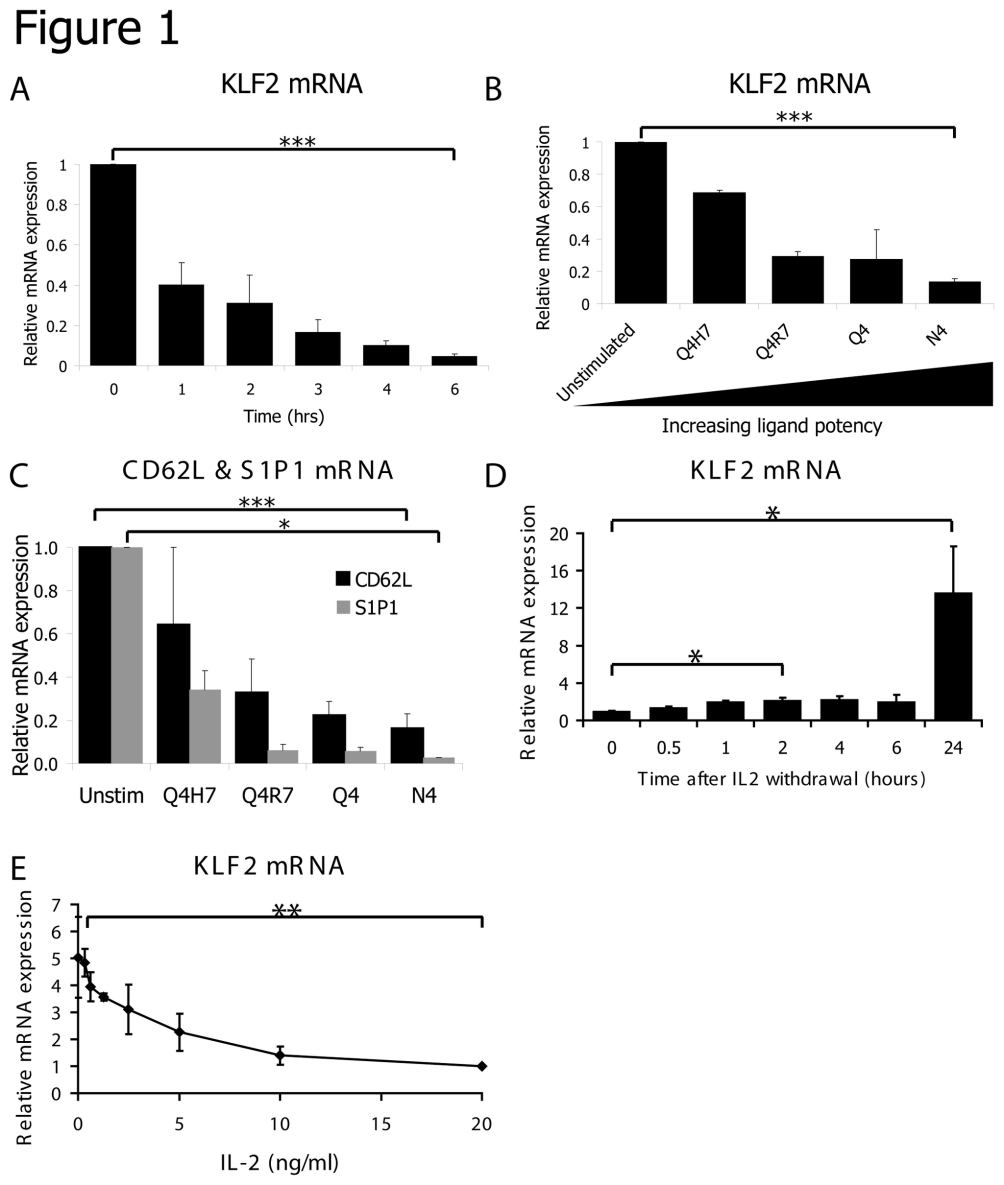
KLF2 expression is regulated by the strength and duration of TCR and cytokine stimulation. (A) Data show relative KLF2 mRNA expression quantified by qRT-PCR in purified naïve CD8 T cells incubated with gp33-41 peptide for the times shown. Results are normalised to untreated naïve cells. (B) KLF2 mRNA in purified naïve OT-1 CD8 T cells incubated with SIIQFEHL (Q4H7) or SIIQFERL (Q4R7), SIIQFEKL (Q4) or SIINFEKL (N4) peptide for 4 hours. Results are normalised to untreated naïve cells. (C) CD62L and S1P1 mRNA in purified naïve OT-1 CD8 T cells incubated with Q4H7, Q4R7, Q4 or N4 peptide for 4 hours. Results are normalised to untreated naïve cells. (D) KLF2 mRNA (normalised to time 0 sample) in T cells activated with gp33-41 peptide for 2 days, cultured in IL-2 for 5 days and then cultured without cytokine for the times shown. (E) KLF2 mRNA (normalised to CTL cultured with 20ng/ml IL-2) in T cells activated with gp33-41 peptide for 2 days, cultured in IL-2 for 5 days and then cultured with the indicated concentrations of IL-2 for 18 hours. All data show mean + SEM of at least 3 independent experiments.

Previous studies have shown that KLF2 is highly expressed in naive T cells but rapidly downregulated at the protein and mRNA level in response to triggering of the T cell antigen receptor[6–8]. The kinetics of this response are shown in Figure 1A. In these experiments, P14-TCR transgenic CD8 T cells were triggered with a peptide of the lymphocytic choriomeningitis virus (LCMV), glycoprotein gp33-41, presented by the MHC class I molecule H-2D^b^. These data show that KLF2 mRNA loss can be detected within 1 hour of triggering the TCR complex and is complete within 6 hours (Figure 1A). The gp33-41 LCMV peptide is a high affinity TCR ligand for the P14 TCR yet during a polyclonal immune response there will be a pool of T cells with diverse affinities for the same antigen. To study how TCR affinity for antigen impacts on KLF2 expression we compared KLF2 levels in OT-1 TCR transgenic CD8 T cells triggered with four altered peptide ligands (APL) derived from the original OT-1 ligand SIINFEKL (N4)[30]. These APL bind equally well to MHC as N4 but differ in their affinity for the OT-1 TCR[30]. Figure 1B shows that the extent of KLF2 downregulation is determined by the potency of the TCR ligand; high affinity ligands strongly reduce KLF2 levels whereas weak TCR ligands are much less effective. The expression of mRNA of two well described KLF2 target genes CD62L and S1P1 is correspondingly decreased by TCR activation with the OT-1 APLs (Figure 1C).

The expression of KLF2 is also modulated by the strength and duration of cytokine signalling. It has been shown previously that IL-2, which promotes CTL differentiation[10,11], maintains low levels of KLF2 in antigen primed T cells[7]. Importantly, IL-2 mediated loss of KLF2 is dependent on continual IL-2 signalling: if CTL are deprived of IL-2 they will re-express KLF2 mRNA within 2 hours and KLF2 mRNA is further increased by 24 hours (Figure 1D). Moreover, the level of KLF2 expression in CTL is also determined by the strength of IL-2 signalling with high concentrations of IL-2 having a stronger suppressive effect on KLF2 expression than low concentrations (Figure 1E). 

**Figure 2:**
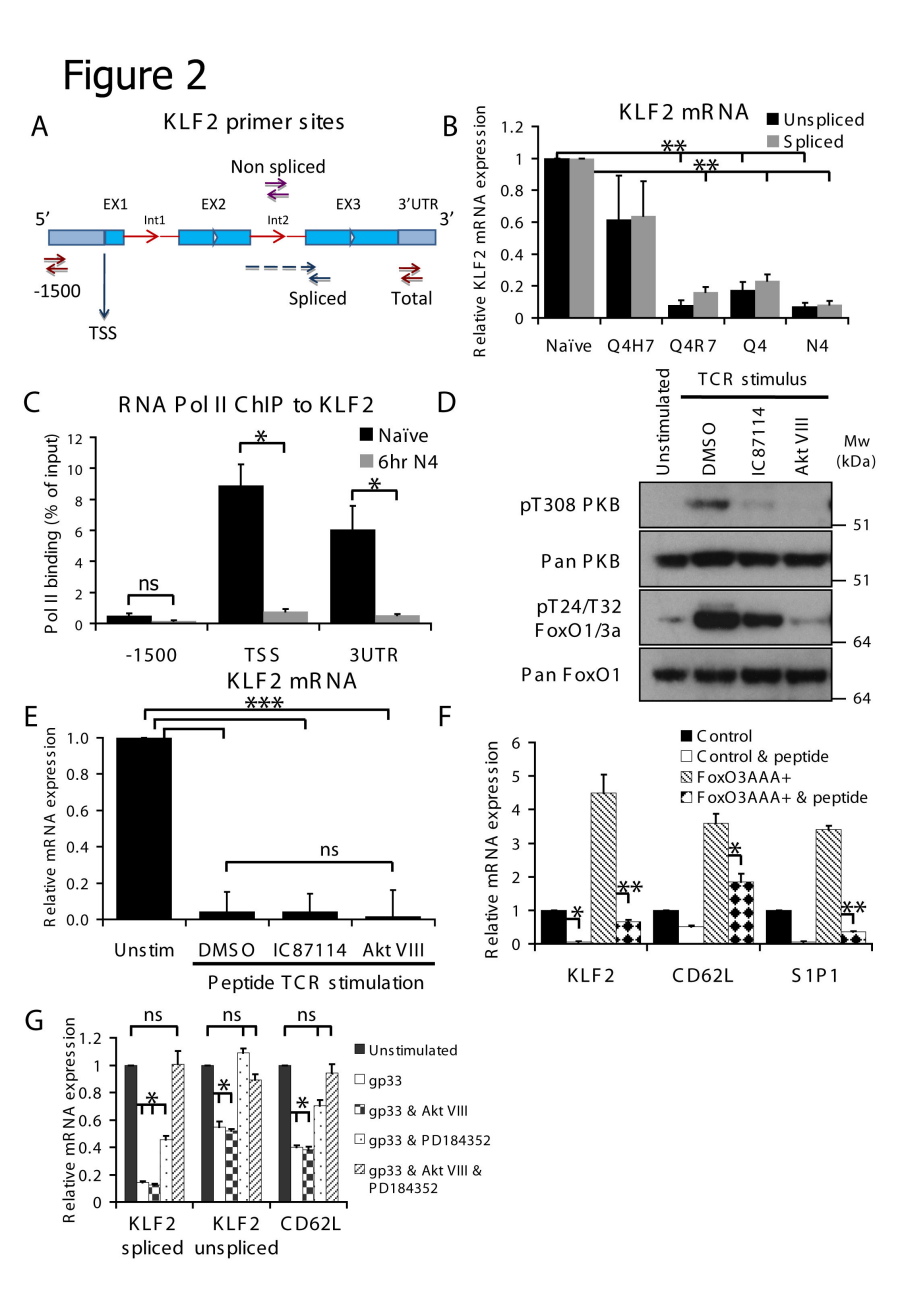
MEK1 and PI3K/PKB activity are required for maximal TCR mediated downregulation of KLF2. (A) Schematic diagram of KLF2 gene indicating binding sites of primers used. (B) Data show binding of RNA polymerase II to the indicated site in the KLF2 gene measured by ChIP assay. Black bars indicate binding in purified naïve OT-1 CD8 T cells, grey bars indicate OT-1 CD8 T cells stimulated with N4 peptide for 6 hours. (C) Spliced and unspliced KLF2 mRNA in purified naïve OT-1 CD8 T cells incubated with Q4H7, Q4R7, Q4 or N4 peptide for 4 hours. Results are normalised to untreated naïve cells. (D) Western blot of lysates from purified naïve OT-1 CD8 T cells incubated with N4 peptide and inhibitors for 4 hours as indicated. (E) KLF2 mRNA in purified naïve P14 CD8 T cells incubated with gp33-41 peptide and inhibitors for 4 hours as indicated. (F) KLF2, CD62L and S1P1 mRNA in FACS purified GFP positive P14 CD8 T cells transduced with control or FoxO3AAA constructs as shown and treated or not with gp33-41 peptide for 4 hours as indicated. (G) CD62L, spliced and unspliced KLF2 mRNA in purified naïve P14 CD8 T cells incubated with gp33 peptide and inhibitors as indicated for 4 hours. Results for each mRNA are normalised to that particular mRNA level in untreated naïve cells – note that comparison of the amount of unspliced or spliced RNA cannot be made. Data in B, C, E, F and G show mean + SEM of at least 3 independent experiments. Data in D are representative of 3 independent experiments.

The loss of KLF2 mRNA in activated T cells could be due to reduced transcription of KLF2 mRNA and/or reflect that TCR triggering decreases KLF2 mRNA stability. To explore these options we compared the impact of TCR engagement on the amount of KLF2 spliced and unspliced RNA. Non-spliced RNA was detected using primers directed to the second intron of the KLF2 transcript whereas spliced RNA was measured using primers spanning the second intron (Figure 2A). Using the OT-1 TCR model we show that both unspliced and spliced KLF2 RNA decreases approximately equally, proportional to baseline, in T cells activated with the different TCR ligands (Figure 2B). The reduction in expression of mature spliced KLF2 mRNA in TCR activated cells could reflect that there is less unspliced KLF2 RNA to be processed in TCR activated T cells compared to naïve T cells, indicating a transcriptional control mechanism. To confirm this finding, we examined the recruitment of RNA polymerase II (Pol II) to the KLF2 gene. We looked at two regions, the transcriptional start site (TSS) and a downstream region within the 3’ untranslated region of the KLF2 gene. Primers directed against a sequence 1500 base pairs upstream of the TSS were used as a negative control. These data (Figure 2C) show that the recruitment of Pol II to the KLF2 locus is strikingly reduced in TCR activated T cells compared to control cells. These data indicate that TCR engagement reduces transcription of the KLF2 gene by dramatically reducing the recruitment of Pol II to the KLF2 locus.

KLF2 expression in naïve T cells is dependent on expression of FoxO1. Hence FoxO1 null naïve T cells do not express KLF2 and have a corresponding loss in expression of CD62L and corresponding defects in cell trafficking[19]. We have shown previously that the ability of IL-2 to downregulate KLF2 expression in CTL is reversed by PI3K or PKB inhibitors[25]. We therefore considered that TCR activation of PKB might mediate TCR induced loss of KLF2. To test this hypothesis purified naïve cells from P14 mice were activated with gp33-41 peptide for 4 hours in the presence or absence of either the PKB inhibitor Akt VIII or the PI3K inhibitor IC87114. In these experiments, IC87114 and Akt VIII inhibited TCR induced phosphorylation of PKB and FoxO1 (Figure 2D). However, surprisingly, both inhibitors failed to prevent the TCR-induced downregulation of KLF2 mRNA (Figure 2E). These results argue that the TCR downregulates expression of KLF2 independently of its ability to control FoxO1 phosphorylation. To directly test this hypothesis we transduced CD8 T cells with the non-phosphorylatable FoxOAAA mutant. Here, all of the PKB sites responsible for FoxO 14-3-3 protein binding and hence the nuclear exclusion seen on TCR activation are mutated to alanine. Thus, the FoxOAAA protein will remain nuclear despite activation of PKB through TCR stimulation. As predicted, expression of FoxOAAA increases expression of KLF2 and its target genes CD62L and S1P1 (Figure 2F). However, the salient observation is that the activation of the TCR still results in a rapid and dramatic downregulation of KLF2 and its target genes. The TCR can thus downregulate expression of KLF2 independently of its ability to activate PKB and induce FoxO phosphorylation.

In Figure 1B we showed that the magnitude of KLF2 downregulation is graded according to the avidity of the different OT-1 TCR peptide ligands. The magnitude of KLF2 downregulation triggered by the different OT1 ligands correlates with the ability of these APLs to activate the p42/p44 MAP kinase (ERK1/2) pathway downstream of the TCR[30]. We therefore explored the role of the MAPK pathway in downregulating KLF2 expression. PD184352 is a well characterised allosteric inhibitor that prevents activation of MEK1, the kinase which phosphorylates and activates ERK1/2[31]. The inhibition of ERK1/2 activation with PD184352 partially blocked TCR mediated downregulation of total KLF2 mRNA and is sufficient to entirely block unspliced KLF2 mRNA downregulation (Figure 2G). Intriguingly, the combination of both MEK and PKB inhibition is required to completely block TCR mediated downregulation of KLF2 and its target gene CD62L (Figure 2G).

### The importance of downregulating KLF2 in T cell activation

**Figure 3:**
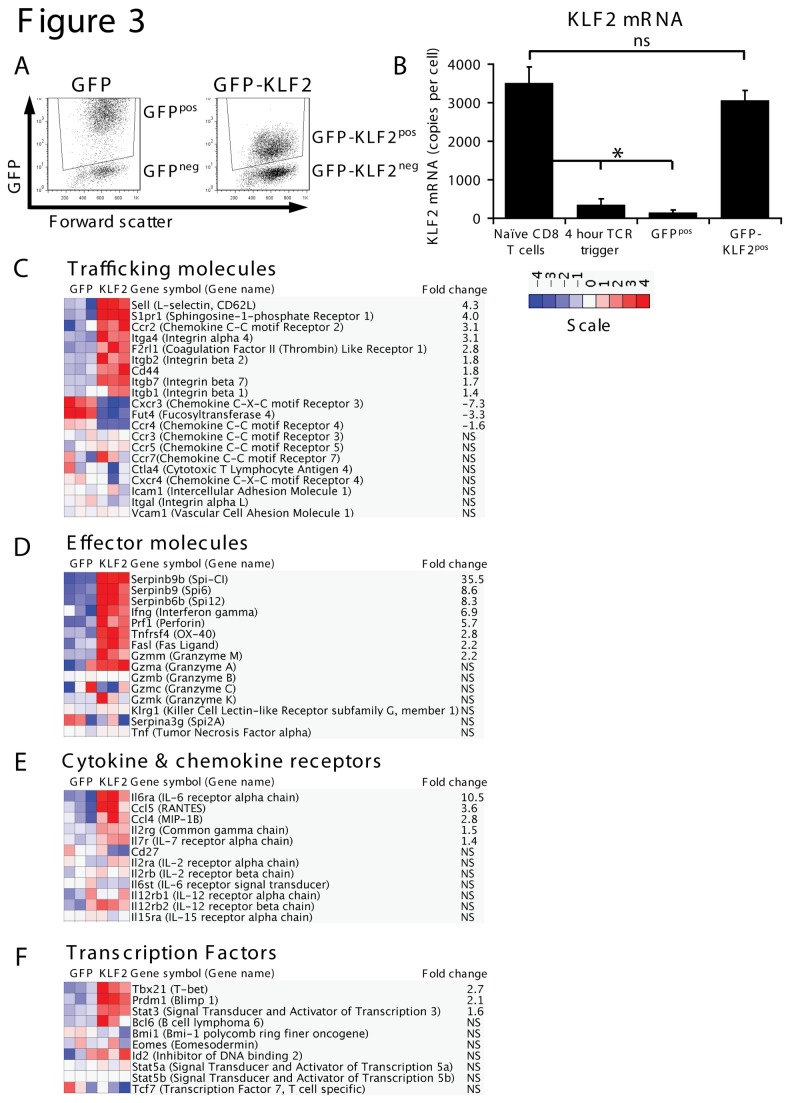
The impact of failing to downregulate KLF2 on the transcriptional programme of TCR activated CD8 T cells. (A) Data show flow cytometric analysis of activated CD8 T cells transduced with either vector (evGFP) or a GFP-KLF2 fusion construct (data representative of 10 independent experiments). In all experiments using these constructs, CD8 T cells were transduced at 18 hours post-activation, washed out of peptide stimulation at 48 hours and then cultured with IL-2 for a further 48-72 hours prior to experimental procedures. (B) Data show KLF2 mRNA expression in purified naïve P14 CD8 T cells, P14 CD8 T cells treated with gp33-41 peptide for 4 hours, GFP positive activated CD8 T cells purified by FACS from activated CD8 T cells transduced with either evGFP or a GFP-KLF2 fusion protein. After reverse transcription, KLF2 mRNA was quantified by qRT-PCR against a standard curve of a KLF2 cDNA and is expressed as mRNA copy number. Data show mean + SEM of 3 independent experiments. (C) Heat map showing gene expression patterns of genes encoding trafficking molecules in GFP^pos^ and GFP-KLF2^pos^ activated CD8 T cells, (D) CD8 T cell effector molecules, (E) cytokine receptors and (F) transcription factors. Heat maps are normalised to depict a 2-fold regulation as significant. Fold changes where shown are statistically significant (p<0.05) and are relative to the GFP^pos^ control.

KLF2 drives expression of CD62L, an adhesion receptor essential for T cell transmigration from the blood into secondary lymphoid tissues, and S1P1, a lysosphingolipid receptor that controls T cell egress from thymic and peripheral lymphoid tissue[14–16]. KLF2 downregulation in response to triggering of antigen and cytokine receptors thus explains why immune activated effector cells lose expression of these key trafficking molecules. However, is KLF2 downregulation required for any other key processes of CD8 T cell activation? Note the impact of KLF2 loss in naïve T cells has been assessed previously[3,4,17] but no experiment has fully explored the importance of the loss of KLF2 expression that accompanies immune activation. The experimental strategy to address this question requires the genetic manipulation of T cells to prevent them downregulating expression of KLF2 in response to immune activation. Accordingly, we decided to use Affymetrix microarray analysis to examine the transcriptional profile of immune activated T cells that were retrovirally transduced to maintain expression of KLF2 mRNA to the levels seen in quiescent naïve T cells (Figure 3A, 3B). In these experiments we compared control activated CD8 T cells versus activated CD8 T cells transduced with a retrovirus encoding GFP-KLF2 fusion protein (Figure 3A). We could analyse four different populations from these experiments; cells transduced with either the GFP-KLF2 construct or with an empty vector GFP construct or those negative cells from the same cultures. This experiment allows an assessment of the importance of KLF2 downregulation for the immune activation of CD8 T cells. For clarity, we refer to these activated CD8 T cell populations throughout the text as GFP-KLF2^pos^, GFP^pos^, GFP-KLF2^neg^ and GFP^neg^ respectively (Figure 3A).

Using this system we compared by microarray activated CD8 T cells with normal downregulation of KLF2 with activated CD8 T cells where KLF2 expression was maintained. Approximately 10000 annotated genes were expressed in activated CD8 T cells and the impact of KLF2 re-expression was to upregulate 492 genes and downregulate 164 genes by at least twofold. The full list of genes regulated by KLF2 is detailed in Table S1. It should be noted that KLF2 is not detected as a regulated gene since the GFP-KLF2 construct lacks the 3’ UTR of KLF2, which is the sequence detected by probes in the microarray. The microarray data corroborated previous data showing that maintaining KLF2 expression prevents activated T cells downregulating expression of CD62L and S1P1 (Figure 3C). To verify these data we analysed GFP-KLF2^pos^ cells for expression of CD62L and S1P1 mRNA (Figure S1A) and CD62L surface protein expression (Figure S1B). The results show that a failure to downregulate KLF2 sustained expression of CD62L and S1P1 in activated CD8 T cells. CD62L expression is frequently used to distinguish terminally differentiated effector CTL from naïve or memory T cells. We therefore examined the impact of maintaining KLF2 expression on other key genes that determine CTL effector function (Figure 3D-F). These analyses found that KLF2 expression did not prevent immune activated T cells acquiring expression of cytolytic effector molecules such as the granzymes (Figure 3D), nor did it prevent expression of key transcription factors that control CTL differentiation such as STAT5 or eomesodermin (Figure 3F). On the contrary, the expression of mRNA encoding IFNγ and perforin were increased by maintaining KLF2 expression (Figure 3D).

Effector T cells can be distinguished by their cytokine receptor profiles. Notably effector T cells downregulate expression of receptors for IL-6 and IL-7 and are CD27 low. They do however express high levels of pro-inflammatory cytokine receptors, notably IL-12 receptors. In this context, maintaining KLF2 expression in activated CD8 T cells had no effect on expression of CD27 or IL-7 and IL-12 receptors but strikingly increased expression of mRNA encoding the IL-6 receptor alpha subunit (Figure 3E). This latter result was verified by qRT-PCR and flow cytometric analysis (Figure S1C, S1D). One other observation was that immune activated T cells that maintained KLF2 expression expressed much higher levels of mRNA encoding the Serpin family of serine protease inhibitors, notably *Serpinb9* (Figure S1E). This is a serine protease inhibitor of granzyme B that controls cytotoxic granule integrity in effector CTL and is expressed at high levels in memory T cells[32,33]. KLF2 upregulation of *Serpinb9* (Spi6) protein was verified by western blot analysis (Figure S1F). Moreover, qRT-PCR analysis confirmed a cell autonomous effect of KLF2 on *Serpinb9* expression (Figure S1E). Collectively, these data show that KLF2 controls expression of a selective portion of the CD8 T cell transcriptional program but persistent KLF2 expression post activation is not sufficient to force the CD8 T cell transcriptional program to remain that of a naïve T cell.

### KLF2 regulates CXCR3 expression in CD8 T cells

**Figure 4:**
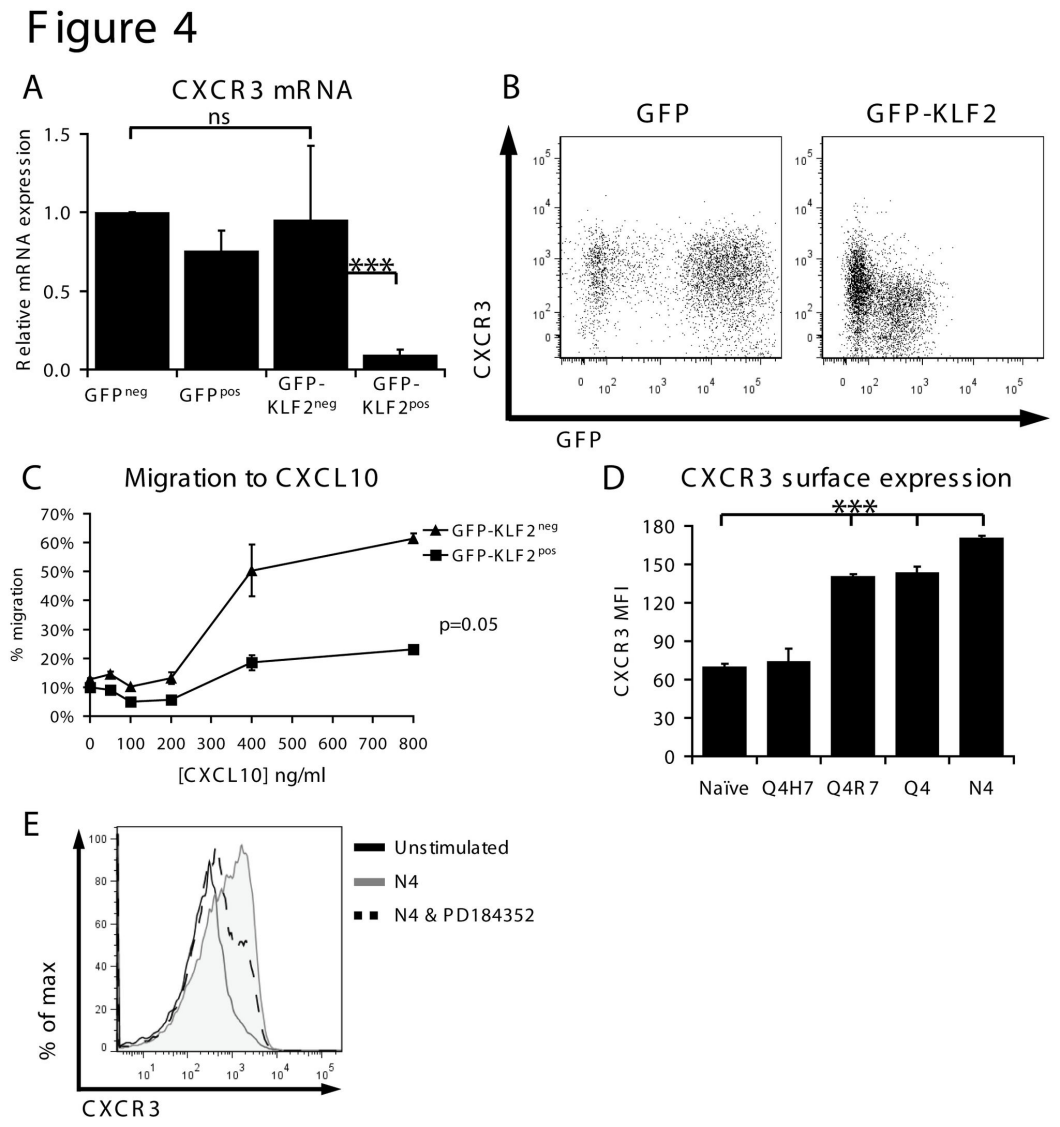
KLF2 represses CXCR3 expression and function. (A) Data show CXCR3 mRNA expression quantified by qRT-PCR in the indicated activated CD8 T cell populations normalised to GFP^neg^. (B) Data show flow cytometric analysis of CXCR3 surface expression and GFP expression in GFP^pos^ or GFP-KLF2^pos^ activated CD8 T cells. (C) GFP-KLF2^neg^ and GFP-KLF2^pos^ activated CD8 T cells were competitively assayed for their ability to migrate to CXCL10. Data shown are the percentage of cells migrating (relative to input controls) at the given CXCL10 concentrations. (D) MFI of CXCR3 quantified by flow cytometry in naïve OT-1 CD8 T cells activated with N4, Q4, Q4R7 or Q4H7 for 24 hours. (E) CXCR3 expression measured by flow cytometry in naïve OT-1 CD8 T cells incubated with N4 peptide and PD184352 for 24 hours as indicated. Data in A, C & D show mean + SEM of 3 independent experiments. Data in B and E representative of 3 independent experiments.

From the microarray analysis, we noted that a failure to downregulate KLF2 prevented upregulation of the inflammatory chemokine receptor CXCR3 in immune activated CD8 T cells (Figure 3C). Previous studies have shown that KLF2 loss can cause thymocytes to express CXCR3 but this is an indirect consequence of unrestrained cytokine production by KLF2 null thymocytes[17]. It was thus striking that KLF2 control of CXCR3 in activated CD8 T cells was cell autonomous. Hence CXCR3 mRNA expression is low in GFP-KLF2^pos^ activated CD8 T cells but high in co-cultured GFP-KLF2^neg^ activated CD8 T cells (Figure 4A). The cell autonomy of KLF2 control of CXCR3 was also confirmed by flow cytometry analysis (Figure 4B) that showed lower surface levels of CXCR3 on GFP-KLF2^pos^ activated CD8 T cells compared to the co-cultured GFP-KLF2^neg^ cells. Importantly, the downregulation of CXCR3 expression caused by KLF2 had a cell autonomous impact on the chemotaxis of activated CD8 T cells in response to the inflammatory chemokine CXCL10. GFP-KLF2^neg^ activated CD8 T cells thus show a good chemotactic response to CXCL10 but this response is lost in GFP-KLF2^pos^ activated CD8 T cells (Figure 4C). These data show that KLF2 downregulation is essential for induction of CXCR3 expression in activated CD8 T cells. Consistent with this idea, the OT-1 APLs induce CXCR3 expression in a manner inversely correlating with KLF2 expression (Figure 4D). Furthermore, the use of a MEK inhibitor, which partially blocks KLF2 downregulation (Figure 2E), is sufficient to reduce TCR mediated induction of CXCR3 (Figure 4E).

### KLF2 downregulation is required for proliferation of activated CD8 T cell proliferation

**Figure 5:**
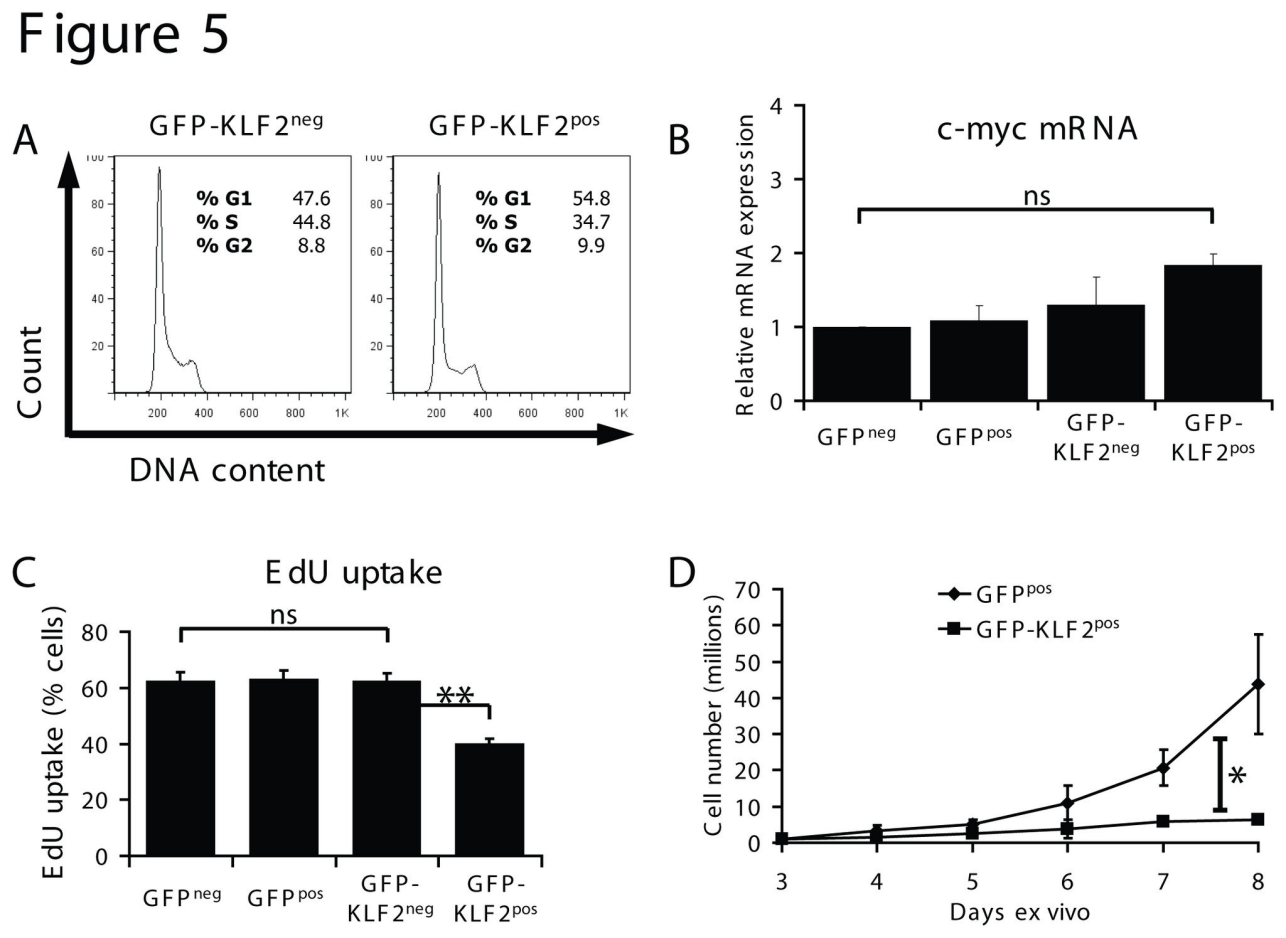
KLF2 re-expression inhibits proliferation but not via *c-myc* repression. (A) Data show the cellular DNA content of GFP-KLF2^neg^ and GFP-KLF2^pos^ activated CD8 T cells. Data are representative of 4 independent experiments. (B) Expression of *c-myc* mRNA in FACS purified activated CD8 T cells quantified by qRT-PCR (data normalised to GFP^neg^ and show mean + SEM of 3 independent experiments). (C) DNA synthesis measured by EdU uptake in the T cell populations shown (data show mean percentage EdU uptake + SEM, n=3). (D) Cell counts of GFP^pos^ and KLF2^pos^ activated CD8 T cells; data shown are mean ± SEM of 3 independent experiments.

Ectopic expression of KLF2 can drive T cell quiescence[12,13,16] but it is unclear whether KLF2 expression levels achieved in previous experiments were in the physiological range[8]. In this context, it has been shown that the loss of KLF2 is not sufficient for T cell proliferation nor is KLF2 essential to initiate T cell quiescence[8]. In the current experiments we maintained KLF2 mRNA expression in immune activated CD8 T cells to levels equivalent to that found in naive T cells (Figure 3B) and found no major differences in the cellular DNA content of GFP-KLF2^pos^ activated CD8 T cells versus GFP-KLF2^neg^ activated CD8 T cells: both populations contained high frequencies of cells in the proliferative S and G2 phases of the cell cycle (Figure 5A). In particular there was no evidence that sustained KLF2 expression caused cell cycle arrest in activated T cells. Previous studies have indicated that KLF2 induces T cell quiescence because KLF2 downregulates expression of *c-myc* and upregulates the cell cycle inhibitor *p21* ^*cip1*^[12,13]. However, the current microarray analysis did not detect any KLF2 regulation of either of these genes (Table S1). Analysis by qRT-PCR (Figure 5B) shows that *c-myc* mRNA levels are comparable in control and GFP-KLF2^pos^ activated CD8 T cells. 

The cell cycle analysis (Figure 5A) shows that immune activated T cells that do not downregulate KLF2 can enter the cell cycle. However, a quantitative assessment of rates of DNA synthesis (Figure 5C) revealed that GFP-KLF2^pos^ activated CD8 T cells have a lower rate of DNA synthesis compared to control activated CD8 T cells that have terminated KLF2 expression. These data predict that activated CD8 T cells with high levels of KLF2 will take longer to complete a cell cycle. To explore this hypothesis we compared the rate of cell doubling of GFP-KLF2^pos^ versus GFP^pos^ activated CD8 T cells. These data revealed that T cells that maintained KLF2 expression had a reduced rate of T cell proliferative expansion (Figure 5D). Further interrogation of the microarray results revealed that maintaining KLF2 expression leads to an increase in expression of the cyclin dependent kinase inhibitors *Cdkn2a, Cdkn2b* and *Cdkn2d*, which could underpin this reduction in clonal expansion. Collectively, the results indicate that KLF2 is not sufficient to drive cells into a quiescent G0/G1 state but KLF2 expression can restrain T cell proliferative expansion. 

### KLF2 has threshold dependent functions

**Figure 6:**
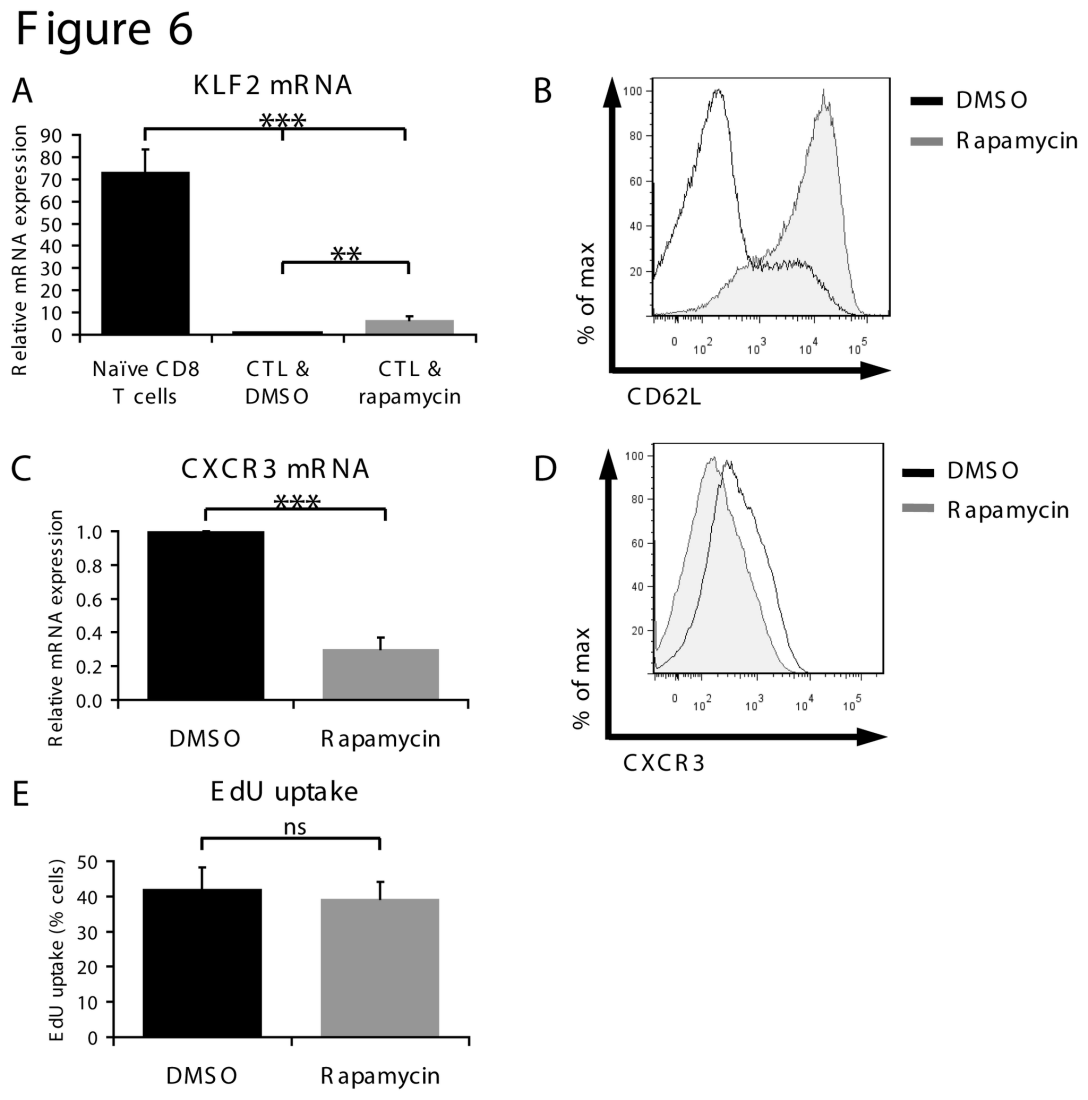
Rapamycin treatment controls trafficking molecules but not DNA synthesis in activated CD8 T cells. (A) Data show expression of KLF2 mRNA determined by qRT-PCR in purified naïve CD8 T cells and T cells activated with gp33-41 peptide for 2 days then cultured with IL-2 plus DMSO or 20nM rapamycin for 5 days. Data are normalised to expression in control T cells and show mean + SEM of 3 independent experiments. (B) CD62L surface expression in CD8 T cells treated as in (A) measured by flow cytometry (data representative of 5 independent experiments). (C) CXCR3 mRNA in CD8 T cells treated as in (A) quantified by qRT-PCR. Data normalised to control T cells and show mean + SEM of 3 independent experiments. (D) CXCR3 surface expression in activated CD8 T cells treated as in (A) measured by flow cytometry, data representative of 3 independent experiments. (E) DNA synthesis measured by EdU uptake in T cells activated with gp33-41 peptide for 2 days then cultured with IL-2 plus either DMSO or rapamycin for 2 days. Data show mean percentage EdU uptake + SEM of 3 independent experiments.

The current results show that maintaining KLF2 expression can restrain CD8 T cell proliferation. However, there are discrepancies in the literature and indeed in the present data that question an obligatory link between the downregulation of KLF2 expression and the induction of T cell proliferation. For example, weak TCR ligands such as the OT-1 peptide Q4H7 do not effectively terminate expression of KLF2 (Figure 1B) yet are able to initiate T cell proliferation[34]. Furthermore, the mTORC1 inhibitor and immunosuppressant rapamycin has been shown to induce KLF2 re-expression and cause re-expression of CD62L and S1P1 in activated CD8 T cells but rapamycin does not inhibit proliferation[7,35]. However, as we have shown, expression of KLF2 in T cells is not simply on or off but can be graded (Figure 1). It has thus been hypothesised that the magnitude of KLF2 expression might dictate the functional outcome in terms of T cell proliferation[8]. In this context although it has been shown that mTOR inhibitors can increase KLF2 expression in activated CD8 T cells it has never been explored whether rapamycin treatment restores KLF2 expression to the levels seen in naïve T cells. The experiment depicted in Figure 6A was designed to explore this issue and compares KLF2 expression in naïve CD8 T cells to activated CD8 T cells cultured in IL-2 in the presence or absence of rapamycin. The data show that rapamycin increases the expression of KLF2 in activated CD8 T cells but to levels far below that seen in naïve cells (Figure 6A). Importantly, mTOR inhibition with rapamycin is sufficient to induce expression of the KLF2 target gene CD62L (Figure 6B) and to repress CXCR3 expression both at the mRNA (Figure 6C) and protein levels (Figure 6D). However, mTOR inhibition with rapamycin does not suppress DNA synthesis in activated CD8 T cells (Figure 6E). 

**Figure 7:**
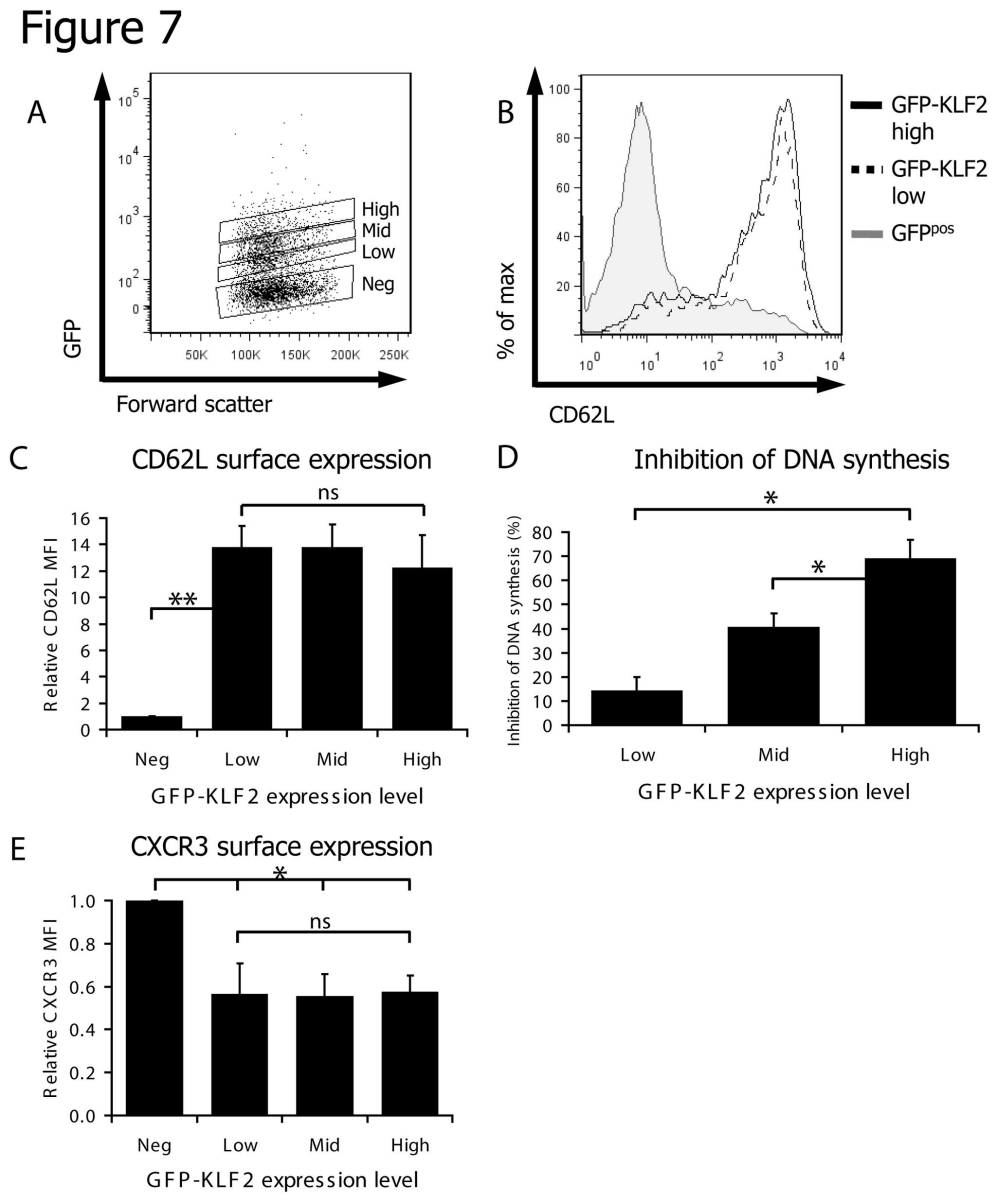
Low KLF2 expression can control trafficking molecules but high KLF2 expression is required to inhibit proliferation. (A) Data show the gating strategy used to identify populations with differential KLF2 expression in GFP-KLF2 transduced activated CD8 T cells. For all experiments P14 CD8 T cells were activated for with gp33-41 peptide and retroviral transduction was performed 18 hours after activation. At 48 hours after the initial activation cells were washed and cultured for a further 3 days with 20ng/ml IL-2. (B) Population expression of CD62L measured by flow cytometry in GFP^pos^, GFP-KLF2^low^ and GFP-KLF2^high^ activated CD8 T cells. (C) CD62L median fluorescence intensities (MFIs) in CD8 T cell populations as indicated. (D) Inhibition of DNA synthesis relative to control GFP-KLF2^neg^ CD8 T cells was quantified for GFP-KLF2^low^, GFP-KLF2^mid^ and GFP-KLF2^high^ CD8 T cells (data show mean + SEM of 3 independent experiments). (E) CXCR3 MFIs in indicated CD8 T cells populations. Data shown in C & E are the mean + SEM of MFIs normalised to GFP^neg^ population from 3 independent experiments.

These results generated a hypothesis that retaining low levels of KLF2 in activated T cells would be sufficient to modulate the CD8 T cell trafficking program yet only high levels of KLF2 similar to those found in naïve T cells would inhibit proliferation. To test this hypothesis we initially examined CD62L expression and DNA synthesis in immune activated CD8 T cells transduced with low, intermediate or high levels of GFP-KLF2 (Figure 7A). Strikingly, the data show that low levels of KLF2 were sufficient to induce maximal expression of CD62L (Figure 7B, 7C) but only very weakly suppressed DNA synthesis (Figure 7D). We also noted that low levels of KLF2 were sufficient to maximally repress expression of CXCR3 (Figure 7E). This argues that KLF2 has different functional consequences depending on its level of expression, with low levels being sufficient to redirect homing and high levels required to inhibit proliferation.

## Discussion

KLF2 is an important transcription factor in T cells and has a key role to control the homing of naïve T cells to secondary lymphoid tissue and to restrain cytokine production in naïve T cells[17]. It is known that KLF2 expression is downregulated in effector T cells. The present results now demonstrate that the loss of KLF2 during an immune response is a graded response determined by the signal strength and duration of both TCR and cytokine signals (Figure 1). Moreover, the loss of KLF2 by immune activated CD8 T cells is reversible and continued downregulation of KLF2 requires continuous signalling to the T cell (Figure 1D-E). Hence, following the removal of strong TCR or cytokine signals from effector T cells, KLF2 is re-expressed. Previous work has shown that in the context of IL-2 signalling the level of KLF2 expression is determined by PI3K/PKB activity[25]. The present results reveal a different pathway of KLF2 regulation in naïve T cells responding to antigen. Hence in the context of TCR triggering the action of both MAP kinases and PI3K/PKB is required to maximally downregulate KLF2 expression (Figure 2). This fine tuning of KLF2 levels in T cells is important because the impact of high versus low KLF2 expression for a T cell is quite different. Hence, a key insight from the present study is that there are different KLF2 thresholds for the regulation of T cell proliferation versus trafficking (Figure 7). KLF2 thus controls an analogue and not a digital response allowing differences in the magnitude of KLF2 expression to fine tune activated CD8 T cells fate. Low levels of KLF2 can thus switch the T cell migratory program whereas only high levels of KLF2 can curb T cell clonal expansion. 

One objective of the present set of experiments was to determine the importance of KLF2 downregulation for T cell activation. The results confirm that maintaining KLF2 expression controls T cell trafficking by maintaining expression of CD62L and S1P1; key molecules that control T cell entry and positioning in secondary lymphoid tissue. However, one other salient observation was that CD8 T cells that maintained KLF2 expression also failed to express the inflammatory chemokine receptor CXCR3 and hence did not acquire the ability to migrate to the CXCR3 ligand CXCL10. The ability of KLF2 to suppress CXCR3 expression was a cell autonomous response (Figure 4) and only required low levels of KLF2 expression (Figures 6C-D, 7E). High affinity OT-1 TCR ligands induced CXCR3 more strongly (Figure 4D) and this effect is mitigated by inhibition of MEK1 (Figure 4E), which is consistent with the idea that higher levels of KLF2 will inhibit CXCR3 expression. The interpretation of our data with regards to KLF2 and CXCR3 is complex for several reasons. The data in Figures 4D-E is purely correlative and a plausible alternative explanation is that the strength of MEK1/ERK signalling controls CXCR3 expression regardless of KLF2. Additionally, we have not been able to prove direct binding of KLF2 to the CXCR3 gene and it is possible that we are observing an indirect effect. Previously published data show that a subset of CD8 memory T cells can co-express CXCR3 and KLF2[17]. However, there is an emerging realisation that the KLF2 transcriptional program is cell context dependent. For example S1P1 is a well validated KLF2 target in T cells but not B cells[36,37]. One possibility is that an unknown co-factor is required for KLF2 to exert its repressive effect on CXCR3 and that this co-factor is regulated in a cell dependent context. The cell autonomy of KLF2 control of CXCR3 expression in TCR triggered CD8 T cells (Figure 4A-C) contrasts with data in thymocytes where KLF2 loss results in upregulation of CXCR3 expression via an indirect mechanism[17]. The expression of CXCR3 in CD8 T cells is not, however, solely controlled by KLF2 repression but requires positive regulation by the transcription factor T-bet[38]. Hence the loss of KLF2 in naïve T cells would thus not be expected to be sufficient to induce CXCR3 expression. In the present experiments KLF2 expression is maintained in activated T cells and although these cells express T-bet there is clearly a dominant repressive effect of KLF2 on CXCR3 expression.

The complexities of the control of CXCR3 expression are relevant because this chemokine receptor mediates effector cell recruitment to sites of inflammation. CXCR3 is also important for the effector CD8 T cell differentiation[39]. Hence targeting signalling pathways that control CXCR3 have high therapeutic potential. For example, CXCR3 directs the trafficking of antigen specific CD8 T cells that induce autoimmune myocarditis[40]. It has moreover been shown that ectopic expression of KLF2 is sufficient to reduce pathogenic responses in a murine model of myocarditis[41]. The present insight that KLF2 can repress CXCR3 expression in activated CD8 T cells provides a molecular explanation for this anti-inflammatory role of KLF2. In a similar context it has been shown that effector CTL treated with rapamycin re-express KLF2 and regain the ability to home to lymph nodes[7]. This change in T cell trafficking was attributed to the re-expression of CD62L and CCR7 in rapamycin treated CTL. However, the present data argue that the ability of rapamycin to re-direct and retain CTL in secondary lymphoid tissue may reflect a combination of effects: the reacquisition of CD62L and CCR7 plus the loss of inflammatory chemokine receptors such as CXCR3. One other intriguing speculation is that the ability of rapamycin to induce KLF2 re-expression might contribute to the recently described ability of this drug to increase the *in vivo* production of memory CD8 T cells by promoting the conversion of effector CD8 T cells into the memory pool[42,43]. It should be emphasised that KLF2 deletion in peripheral T cells does not seem to prevent the development of persistent Ag-specific memory-like cells in response to viruses[17]. Nevertheless, the switch in trafficking of antigen primed T cells in response to rapamycin would favour memory cell production. Moreover, the ability of KLF2 to suppress expression of CXCR3 would also favour memory cell generation. Hence CXCR3 null CD8 T cells respond to immune stimulation by preferentially differentiating to memory cells rather than effector cells[39]. Consistent with our work on the regulation of KLF2 and CXCR3, recent work has shown that the strength of the TCR signal controls the differentiation fate of CD8 T cells and their trafficking properties[44]. Thus, strong signals lead to terminally differentiated effector CD8 T cells that migrate to inflamed peripheral tissues whereas weak signals result in CD8 T cells that home to lymph nodes.

In conclusion, we show herein that KLF2 expression in T cells can be graded dependent upon the strength and duration of immune stimulation. KLF2 downregulation is not solely controlled by the strength of PKB activity in T cells but is also regulated by MAP kinases. The downregulation of KLF2 in response to immune activation is shown to be important for CD8 T cell activation. KLF2 is not strictly a quiescence factor capable of terminating cell cycle progression in immune activated T cells but rather a cellular brake that can restrain T cell proliferative expansion. A key insight is that different levels of KLF2 cause a different functional response in T cells. Rather than acting as a simple on/off switch, KLF2 thus has distinct functions across its gradient of physiological expression.

## Supporting Information

Figure S1 **KLF2 increases expression of S1P1, CD62L, Serpinb9 and Il6ra in CTL.** (A) Data show expression level of S1P1 and CD62L mRNA quantified by qRT-PCR in FACS purified activated CD8 T cells (normalised to GFPpos CD8 T cells). Data show mean + SEM of 5 independent experiments. (B) Data show flow cytometric analysis of CD62L surface expression in activated CD8 T cells populations transduced with either evGFP or the GFP-KLF2 fusion protein (data representative of 3 independent experiments). (C) Il6ra mRNA in FACS purified activated CD8 T cells quantified by qRT-PCR (data normalised to GFPneg and show mean + SEM of 3 independent experiments). (D) IL-6 receptor alpha chain (CD126) surface expression in GFPpos or GFP-KLF2pos activated CD8 T cells measured by flow cytometry, data representative of 3 independent experiments. (E) Serpinb9 mRNA in FACS purified activated CD8 T cells quantified by qRT-PCR (data normalised to GFPneg and show mean + SEM of 3 independent experiments). (F) Spi6 protein expression in FACS purified activated CD8 T cells determined by Western blotting, data representative of 3 independent experiments.
(TIF)

Table S1 **KLF2 regulated genes in activated CD8 T cells.** Table of genes that are statistically significantly different and regulated by at least twofold from the microarray comparison of activated CD8 T cells transduced with a GFP-KLF2 construct and a control evGFP construct. CD8 T cells were transduced at 18 hours post-activation, washed out of peptide stimulation at 48 hours and then cultured with IL-2 for a further 72 hours prior to selection of GFP positive cells by FACS and RNA extraction.
(XLS)
