# Supplementary figures and images for: The Impact of KLF2 Modulation on the Transcriptional Program and Function of CD8 T Cells
Source: PLoS One. 2013 Oct 14;8(10):e77537. doi: 10.1371/journal.pone.0077537 (PMC3796494; doi:10.1371/journal.pone.0077537)

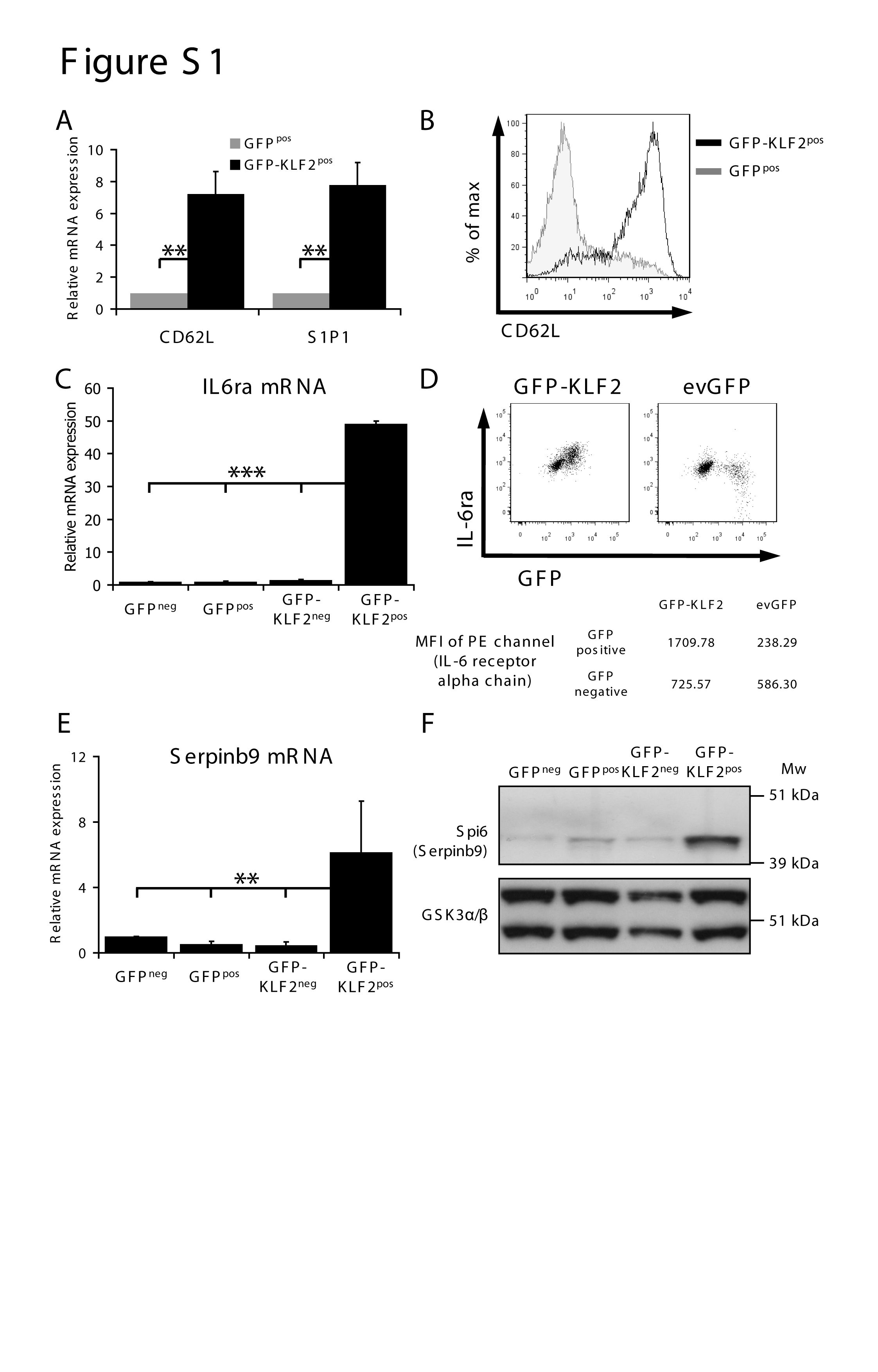

Supplement: Figure S1 — KLF2 increases expression of S1P1, CD62L, Serpinb9 and Il6ra in CTL. (A) Data show expression level of S1P1 and CD62L mRNA quantified by qRT-PCR in FACS purified activated CD8 T cells (normalised to GFPpos CD8 T cells). Data show mean + SEM of 5 independent experiments. (B) Data show flow cytometric analysis of CD62L surface expression in activated CD8 T cells populations transduced with either evGFP or the GFP-KLF2 fusion protein (data representative of 3 independent experiments). (C) Il6ra mRNA in FACS purified activated CD8 T cells quantified by qRT-PCR (data normalised to GFPneg and show mean + SEM of 3 independent experiments). (D) IL-6 receptor alpha chain (CD126) surface expression in GFPpos or GFP-KLF2pos activated CD8 T cells measured by flow cytometry, data representative of 3 independent experiments. (E) Serpinb9 mRNA in FACS purified activated CD8 T cells quantified by qRT-PCR (data normalised to GFPneg and show mean + SEM of 3 independent experiments). (F) Spi6 protein expression in FACS purified activated CD8 T cells determined by Western blotting, data representative of 3 independent experiments. (TIF) [file pone.0077537.s001.tif]
